# Effect of Auditory and Tactile Stimulation on the Level of Consciousness and Physiological Parameters in ICU Patients With Altered Consciousness

**DOI:** 10.1155/ccrp/8835519

**Published:** 2026-02-25

**Authors:** Vinay Kumari, Choki Dema, Jyoti Sarin

**Affiliations:** ^1^ Department of Medical Surgical Nursing, M.M College of Nursing, Maharishi Markandeshwar (Deemed to be University), Mullana, Ambala, Haryana, India, mmumullana.org; ^2^ Department of Medical Surgical Nursing, Arura Academy of Health Sciences, Khesar Gyalpo University of Medical Sciences of Bhutan, Thimphu, Bhutan, kgumsb.edu.bt; ^3^ Department of Child Health Nursing, M.M College of Nursing, Maharishi Markandeshwar (Deemed to Be University), Mullana, Ambala, Haryana, India, mmumullana.org

**Keywords:** consciousness, intensive care unit, sensory deprivation, sensory integration, vital signs

## Abstract

**Objective:**

To evaluate the effectiveness of multisensory stimulation in enhancing consciousness levels and physiological parameters among critically ill patients in the ICU.

**Methods:**

A quasi‐experimental, nonequivalent control group pretest–post‐test design was employed. Seventy‐one patients with Glasgow Coma scale (GCS) scores below 13 were recruited through convenience sampling. Participants were divided into an experimental group (*n* = 34), which received multisensory stimulation twice daily for 7 days, and a comparison group (*n* = 37), which received standard ICU care. Physiological indicators (heart rate, respiration rate, blood pressure, temperature, SpO_2_, and glucose) and consciousness levels (GCS) were determined before and after the intervention. Data were analyzed using SPSS version 20.0, with statistical significance established at *p* ≤ 0.05.

**Result:**

Experimental and comparison groups showed significant differences in consciousness from day 4 to 7 with higher GCS scores in the experimental group. Repeated measures ANOVA demonstrated improvement in GCS scores from the evening of day 2 till day 7 (*p* = 0.05). In contrast, the comparison group experienced a higher incidence of physiological adverse events such as tachycardia and bradycardia.

**Conclusion:**

Multisensory stimulation is a safe and effective method to enhance consciousness in critically ill patients without inducing adverse physiological effects. Early integration of MSS into ICU protocols is recommended. Further research is needed to explore MSS’s efficacy across diverse medical conditions.

## 1. Introduction

Extended ICU stays, in which patients have reduced sensory input, can raise the risk of sensory deprivation, which may then affect central nervous system function. The brain is involved in controlling a person’s state of consciousness, with structures including the brainstem, thalamus, and cerebral cortex being necessary to ensure awareness. Damage to these regions can result in varying levels of unconsciousness, ranging from a profound coma to persistent unresponsiveness and a limited consciousness response [1]. Coma is a severe neurological condition characterized by prolonged impairment of wakefulness and awareness, where the patient remains unresponsive to external stimuli. In contrast, a vegetative state involves the preservation of basic autonomic functions, including cardiovascular regulation, respiration, and temperature control, alongside the partial restoration of the sleep–wake cycle, often marked by spontaneous eye‐opening [2, 3].

The minimally conscious state is characterized by spasmodic but goal‐directed behavioral responses, demonstrating partial consciousness, like obeying commands, gestural communication, or following objects with the eyes, expressing some degree [4].

To reduce the impact of sensory deprivation, multisensory stimulation may be used as a therapeutic approach to improve brain function and foster neuroplasticity. The structured and systematic delivery of multisensory stimuli in unconscious patients has the potential to facilitate neural reorganization and support the restoration of functional brain activity, contributing to improved cognitive and physiological recovery. The coma arousal and brain plasticity theories assume that the brain has an intrinsic adaptive capability allowing it to create new pathways of neurons by bypassing damaged ones eventually forms new neural pathways by circumventing damaged areas ultimately re‐establishing communication with healthy brain cells. This adaptive process can be stimulated and accelerated through enhanced environmental sensory input [5, 6].

Li et al. found that sensory stimulation causes activity in the brain via activating the reticular activating system (RAS). The activation of the RAS causes the release of norepinephrine at nerve terminals, which contributes to higher stimulation and awareness levels. Furthermore, repeated and varied sensory input has been shown to promote dendritic growth and synaptic connection in the damaged nervous system, hence promoting neurological healing [7].

The advantages of multisensory stimulations are evidenced by studies showing that such stimulation can have a positive effect on patients′ responsiveness and induce neurocognitive recovery in various neurological disorders, such as auditory, tactile, visual, olfactory, gustatory (taste), and kinesthetic [8]. Extensive research has demonstrated that sensory stimulation serves as an effective therapeutic approach, capable of activating the RAS. This activation supports neural reorganization by facilitating the formation of new neural pathways, contributing to brain function, restoration, and cognitive recovery. Emerging research suggests that multisensory stimulation may serve as a nonpharmacological intervention capable of enhancing neural networks in the brain and facilitating the recovery of consciousness in these patients. Multisensory stimulation is about stimulating more than one sense like sight, sound, touch, smell, and taste, either at the same time or in succession. Evidence evinces its beneficial effect on patient responsiveness and neurocognitive recovery, especially in neurological disorders impacting consciousness. Through stimulating different sensory pathways, it helps improve cognitive functions and rehabilitation processes [9, 10].

## 2. Methods

### 2.1. Study Design and Patients

**FIGURE 1:**
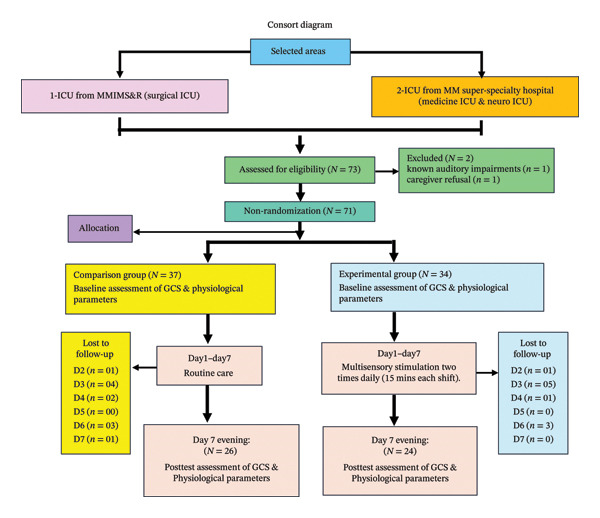
Consort diagram.

A quasi‐experimental, nonequivalent control group pretest–post‐test research design was employed from December 2024 to February 2025 to gather data from 71 patients having altered consciousness (Glasgow Coma scale [GCS] score < 13) and admitted under Medicine ICU & Neuro ICU of MM Super Specialty and MMIMS&R Hospital, Mullana, Ambala. The study population meeting inclusion criterion were nonrandomly divided into experimental group (*n* = 34) and comparison group (*n* = 37), respectively (Figure 1). The patient’s caregivers gave written consent prior to the study commencement. Ethical clearance for the study was provided by the Institutional Ethical Committee of MM (Deemed to be University), Mullana, Ambala.

### 2.2. Patient Selection

#### 2.2.1. Inclusion Criteria

The study included ICU patients with altered consciousness and a GCS score of < 13. Participants must be at least 18 years old and have authorization from a family member or legal guardian.

#### 2.2.2. Exclusion Criteria

The study excluded patients with known auditory problems and those without a caretaker.

### 2.3. Sample Size and Power Calculation

A power analysis was conducted using the post‐test GCS scores from the previous study [11] to determine the appropriate sample size for this study. The estimated effect size was 1.8 with a power of 0.80. Based on this analysis, the recommended sample size for each group was 17, resulting in a total of 34 eligible patients with altered consciousness.

### 2.4. Data Collection

The data collection instruments used to collect data were GCS scale and physiological parameters scale. The sociodemographic characteristics included age, gender, marital status, education status, occupation, religion, monthly income (₹), history of drug usage, family type, and caregiver–patient connection. Clinical variables included primary diagnosis, comorbidities, and GCS score. Physiological parameters measured were systolic and diastolic blood pressure, respiratory rate, heart rate, body temperature, oxygen saturation (SpO_2_), and random blood glucose levels.

For measurement of level of consciousness GCS by Royal College of Physicians and Surgeons of Glasgow (https://www.glasgowcomascale.org/) was used. It assesses three dimensions of responsiveness (EVM) from lowest 3 to highest 15. The content validity of the instrument and intervention was determined by sending the tool along with a brief summary of the intervention to seven experts.

Content validity of the sociodemographic variables was SCVI‐0.95 and ICVI‐0.88–1. Content validity of the clinical variables was SCVI‐0.82 and ICVI‐0.75–0.88. Content validity of the physiological parameters was SCVI‐0.95 and ICVI‐0.63–1. The reliability of the GCS was assessed through inter‐rater reliability, conducted by the researcher and the expert from Medical‐Surgical Nursing Department who independently scored the patient’s level of consciousness to ensure consistency and agreement in their assessments. The reliability of each component was Eyes = 1, Verbal = 0.9, and Motor = 0.8. The total GCS reliability coefficient was 0.99 within the acceptance range of 0.7–1.0.

First, data were gathered from the comparison group and then experimental group in order to avoid the contamination. The researcher reviewed the patient’s charts and documents 24 h after the patient was admitted in the targeted ICUs to document the sociodemography characteristics and clinical variables related to the nature of the study.

A pretest was done on the participant using the GCS and physiological parameter measurements to determine the baseline readings. No Intervention of Multisensory Stimulation was provided to the comparison group. The comparison group got normal ICU regular care, including daily vital monitoring, oxygenation and ventilator support, continuous monitoring of fluid and electrolyte balance, fluid intake and output charting, pharmaceutical management, and dietary assistance. GCS scores and physiological data were recorded four times per day: twice in the morning and twice in the evening, each for 15 min. Post‐tests were administered on day 7 and/or the day of discharge to evaluate the changes.

The experimental group received a pretest on the first day of the study in the form of an assessment utilizing the GCS and physiological measures to create baseline readings. The experimental group received multisensory stimulation twice daily, once in the morning by a selected caregiver who was closest to the patient, and once in the evening by the researcher. Multisensory stimulation was provided as per the hospital’s established visiting hours, from 11:00 a.m. to 12:00 noon in the morning and 4:00 p.m. to 5:00 p.m. This multisensory stimulation was provided for seven consecutive days. Each session lasted 15 min, divided into three sets of 5 min each, with a 1‐min break between the sets. The first set comprised a structured auditory stimulation personalized to individual patients’ need for the best possible outcomes. In the auditory stimulation, the family member provided orientation to the patient by sharing information about the time, month, and day of the week, weather, the patient’s current setting, ongoing treatments or procedures, and details about the family and financial situation. After a minute of break, tactile stimulation was implemented. This included lightly stroking the patient’s forehead; offering a gentle scalp massage; holding the patient’s hand or touching their arm; and massaging the palms, fingers, soles, and toes in a circular motion to stimulate those areas. The family member also applied gentle pressure along the forearms and calves and carefully moved the patient’s limbs or adjusted their position to help prevent stiffness and enhance comfort. After a 1‐min break, the caregiver repeated the auditory and tactile engagement from the first two sessions.

On day 7, post‐test of the consciousness level and physiological parameters was assessed and recorded in both the experimental and comparison groups. A total of 22 mortality cases were recorded by the 7th day, with 11 cases in each of the comparison and experimental groups.

The pretest value for GCS and physiological parameters was determined by the first measurement recorded at 11:00 a.m. The post‐test value was obtained by averaging the last two measurements taken in the evening.

### 2.5. Statistical Analysis

Data were analyzed using SPSS version 20.0. Descriptive statistics were used to summarize demographic and clinical characteristics. Inferential statistics included chi‐square tests for categorical variables, independent *t*‐tests for continuous variables, and repeated measures ANOVA to assess changes in GCS scores over time. Statistical significance was set at *p* ≤ 0.05. Effect sizes were reported using Cohen’s d.

## 3. Result

### 3.1. Patients’ Characteristics

Most patients in both groups were between the ages of 60 and 87 years as indicated in Table 1. More than half of the patients in both the experimental and comparison groups were male, accounting for 55.8% and 62.2% of the participants, respectively. There were no statistically significant differences between the experimental and comparison groups in terms of age, gender, marital status, education, occupation, religion, income, substance use history, or primary diagnosis (*p* > 0.05), confirming baseline homogeneity (Tables 1 and 2).

**TABLE 1:** Comparison of experimental and comparison groups in terms of sample characteristics.

S. No.	Variables	Comparison group (*n* = 37)	Experimental group (*n* = 34)	(*χ* ^2^)	df	*p* value
		*f* (%)	*f* (%)			
1	Age					
1.1	18–29	1 (2.7%)	5 (14.7%)	4.63	3	0.20
1.2	30–44	7 (18.9%)	3 (8.8%)			
1.3	45–59	8 (21.6%)	9 (26.5%)			
1.4	60–87	21 (56.8%)	17 (50.0%)			

2	Gender					
2.1	Male	23 (62.2%)	19 (55.9%)	0.29	1	0.59
2.2	Female	14 (37.8%)	15 (44.1%)			

3	Marital status					
3.1	Married	30 (81.1%)	28 (82.4%)	3.90	2	0.14
3.2	Unmarried	2 (5.4%)	5 (14.7%)			
3.3	Divorced/separated	5 (13.5%)	1 (2.9%)			

4	Educational status					
4.1	No formal education	5 (13.5%)	9 (26.5%)	7.33	4	0.12
4.1	Primary	11 (29.7%)	8 (23.5%)			
4.3	Up to 10th standard	10 (27.0%)	3 (8.8%)			
4.4	Up to higher secondary	9 (24.3%)	8 (23.5%)			
4.5	Graduate and above	2 (5.4%)	6 (17.6%)			

5	Occupation					
5.1	Unemployed	6 (16.2%)	4 (11.8%)	2.22	4	0.69
5.2	Self‐employed	9 (24.3%)	10 (29.4%)			
5.3	Government job	8 (21.6%)	4 (11.8%)			
5.4	Private job	3 (8.1%)	2 (5.9%)			
5.5	Home makers	11 (29.7%)	14 (41.2%)			

6	Religion					
6.1	Hindu	31 (83.8%)	25 (73.5%)	2.67	3	0.45
6.2	Muslim	1 (2.7%)	2 (5.9%)			
6.3	Sikh	4 (10.8%)	7 (20.6%)			
6.4	Christian	1 (2.7%)	0 (0.0%)			

7	Income/month (₹)					
7.1	< 5000	8 (21.6%)	6 (17.6%)	3.99	4	0.41
7.2	5001–10,000	2 (5.4%)	2 (5.9%)			
7.3	10,001–15,000	4 (10.8%)	10 (29.4%)			
7.4	15,001–20,000	10 (27.0%)	7 (20.6%)			
7.5	> 20,000	13 (35.1%)	9 (26.5%)			

8	History of Substance abuse					
8.1	None	20 (54.1%)	19 (55.9%)	1.22	3	0.75
8.2	Alcohol	9 (24.3%)	7 (20.6%)			
8.3	Smoking	4 (10.8%)	6 (17.6%)			
8.4	Both	4 (10.8%)	2 (5.9%)			

9	Family Types					
9.1	Nuclear	9 (24.3%)	6 (17.6%)	0.47	1	0.49
9.2	Joint	28 (75.7%)	28 (82.4%)			

10	Caregiver’s relation					
10.1	Spouse	10 (27.0%)	0 (0.0%)	13.48	3	0.004^∗^
10.2	Son	5 (13.5%)	13 (38.2%)			
10.3	Daughter	5 (13.5%)	5 (14.7%)			
10.4	Relatives	17 (45.9%)	16 (47.1%)			

*Note: N* = 71, ^NS^Not significant (*p* > 0.05).

^∗^Significant (*p* ≤ 0.05).

**TABLE 2:** Comparison of experimental group and comparison group in terms of clinical variables.

S. No.	Variables	Comparison group (*n* = 37)	Experimental group (*n* = 34)	Chi square (*χ* ^2^)	df	*p* value
1	Primary Diagnosis					
1.1	Cardio‐thoracic	7 (18.9%)	6 (17.6%)	4.02	3	0.26^NS^
1.2	Renal	7 (18.9%)	8 (23.5%)			
1.3	Endocrine	1 (2.7%)	5 (14.7%)			
1.4	Neurology	22 (59.5%)	15 (44.1%)			

2	Comorbid illness					
2.1	Yes	30 (81.1%)	25 (73.5%)	0.58	1	0.45^NS^
2.2	No	7 (18.9%)	9 (26.5%)			

3	Previous surgeries					
3.1	Yes	3 (8.1%)	4 (11.8%)	0.27	1	0.61^NS^
3.2	No	34 (91.9%)	30 (88.2%)			

4	GCS					
4.1	3–8	24 (64.9%)	15 (44.1%)	3.08	1	0.08^NS^
4.2	9–12	13 (35.1%)	19 (55.9%)			

*Note: N* = 71. ^NS^Not significant (*p* > 0.05).

A high percentage of participants in the comparison group (59.5%) and the experimental group (44.1%) had been diagnosed with neurological disorders like subdural hematoma (SDH), cerebrovascular accident (CVA), and meningitis. Besides neurological diseases, there were patients admitted under cardiothoracic (18.9% vs. 17.6%) and renal diseases (18.9% vs. 23.5%) in the experimental vs comparison group as reported in Table 2.

Table 3 shows mean GCS scores on admission. The experimental group had a mean GCS score of 8.09 ± 3.14 on day 1 before receiving auditory and tactile stimulations, whereas the comparison group had a mean GCS score of 6.62 ± 3.51. There was no significant difference in mean GCS scores between the two groups (*p* = 0.07). Nonetheless, a notable rise in the level of consciousness was noted from day four after receiving multisensory stimulation (*p* = 0.01) illustrated by Tables 4 and 5. The rise continued to be statistically significant from 4 days to 7 days (*p* = 0.001) with an effect size of 1.085 illustrated by Table 6.

**TABLE 3:** Comparison of level of consciousness (GCS) before administration of multisensory stimulation in experimental group and comparison group.

Level of consciousness (GCS)	Comparison (*n* = 37)	Experimental (*n* = 34)	*M*_*D*_	SE_MD_	*t*	df	*p*value
	Mean ± SD	Mean ± SD					
Day‐1	6.62 ± 3.51	8.09 ± 3.14	−1.47	0.79	−1.85	69	0.07^NS^

*Note:* Pretest GCS is the 1st reading of GCS in the morning on day 1, *N* = 71, ^NS^Not significant (*p* > 0.05).

**TABLE 4:** Day‐wise comparison of mean GCS after administration of multisensory stimulation in the morning in experimental and comparison groups.

Day	Group		*M*_*D*_	SE_MD_	*t*	Df	*p* value
	Comparison mean ± SD	Experimental mean ± SD					
*Day-1*							
C (*n* = 37), E (*n* = 34)	6.62 ± 3.51	8.09 ± 3.14	−1.47	0.79	−1.85	69	0.07^NS^

*Day-2*							
C (*n* = 36), E (*n* = 33)	6.53 ± 3.66	8.18 ± 3.36	−1.65	0.85	−1.95	67	0.06^NS^

*Day-3*							
C (*n* = 32), E (*n* = 28)	9.41 ± 8.66	12.11 ± 3.79	−2.70	1.77	−1.53	58	0.13^NS^

*Day-4*							
C (*n* = 30), E (*n* = 27)	9.10 ± 4.51	12.89 ± 3.53	−3.79	1.08	−3.51	55	0.001^∗^

*Day-5*							
C (*n* = 30), E (*n* = 27)	9.73 ± 4.79	13.30 ± 3.49	−3.56	1.12	−3.17	55	0.002^∗^

*Day-6*							
C (*n* = 27), E (*n* = 24)	8.63 ± 5.42	14.08 ± 2.54	−5.45	1.21	−4.51	49	0.001^∗^

*Day-7*							
C (*n* = 26), E (*n* = 24)	8.92 ± 5.18	13.67 ± 3.39	−4.74	1.25	−3.79	48	0.001^∗^

*Note:* ^NS^Not significant (*p* > 0.05).

^∗^Significant (*p* ≤ 0.05).

**TABLE 5:** Comparison of mean GCS after administration of multisensory stimulation in the evening in experimental and comparison groups.

Day	Group		*M*_*D*_	SE_MD_	*t*	df	*p* value
	Comparison mean ± SD	Experimental mean ± SD					
*Day-1*							
C (*n* = 37), E (*n* = 34)	6.70 ± 3.48	8.09 ± 3.14	−1.39	0.79	−1.76	69	0.08^NS^

*Day-2*							
C (*n* = 36), E (*n* = 33)	6.62 ± 3.71	8.61 ± 3.68	−2.41	0.85	−2.84	67	0.06^NS^

*Day-3*							
C (*n* = 32), E (*n* = 28)	8.31 ± 4.45	13.71 ± 8.76	−5.40	1.76	−3.07	58	0.003^∗^

*Day-4*							
C (*n* = 30), E (*n* = 27)	9.23 ± 4.45	13.04 ± 3.49	−3.80	1.07	−3.56	55	0.001^∗^

*Day-5*							
C (*n* = 30), E (*n* = 27)	9.87 ± 4.69	13.30 ± 3.49	−3.43	1.11	−3.09	55	0.003^∗^

*Day-6*							
C (*n* = 27), E (*n* = 24)	8.63 ± 5.42	14.17 ± 2.53	−5.54	1.21	−4.58	48	0.001^∗^

*Note:* ^NS^Not significant (*p* > 0.05).

^∗^Significant (*p* ≤ 0.05).

**TABLE 6:** Comparison of post‐test GCS in the experimental group and comparison group.

Level of consciousness (GCS)	Comparison (*n* = 26)	Experimental (*n* = 24)	*M*_*D*_	SE_MD_	*t*	df	*p* value	Effect size (Cohen’s d)
	Mean ± SD	Mean ± SD						
Day‐7	8.92 ± 5.18	13.67 ± 3.39	−1.74	1.25	−3.79	48	0.001^∗^	1.085

*Note:* Day‐7 GCS denotes the average GCS score recorded at 15‐min interval in the evening of day 7, *N* = 50, ^NS^Not significant (*p* > 0.05).

^∗^Significant (*p* ≤ 0.05).

Repeated measures ANOVA revealed significant changes in GCS scores in the experimental group (*F* = 651.32, *p* = 0.001) and in the comparison group (*F* = 142.58, *p* = 0.001) at the 0.05 level of significance illustrated by Table 7. Post hoc analysis following repeated measures ANOVA shown in Table 8, demonstrates a significant improvement in GCS scores following multisensory stimulation, with changes observed from the evening of day 2 in the experimental group (*p* = 0.05). However, a significant improvement was also noted in the comparision group from the morning of day 3*p* = 0.001) as shown in Tables 9 and 10.

**TABLE 7:** RM‐ANOVA showing comparison of consciousness (GCS) before and after administration of multisensory stimulations from day 1 to day 7 in the experimental group.

Group	Time periods	Mean ± SD	*F* value	*p* value
Experimental	Pretest	9.17 ± 2.48	651.32	0.001^∗^
	Day‐1 (M)	9.17 ± 2.48		
	Day‐1 (E)	9.17 ± 2.48		
	Day‐2 (M)	9.42 ± 2.60		
	Day‐2 (E)	10.00 ± 2.89		
	Day‐3 (M)	12.46 ± 3.67		
	Day‐3 (E)	14.33 ± 9.20		
	Day‐4 (M)	13.04 ± 3.32		
	Day‐4 (E)	13.21 ± 3.26		
	Day‐5 (M)	13.50 ± 3.24		
	Day‐5 (E)	13.50 ± 3.24		
	Day‐6 (M)	14.08 ± 2.54		
	Day‐6 (E)	14.17 ± 2.53		
	Day‐7 (M)	13.67 ± 3.40		
	Post‐test	13.67 ± 3.40		

*Note:* From day 1 to day 7, the M&E value represents the average of GCS recorded at an interval of 15 min (Sample size for RMANOVA is 24 as only 24 patients had GCS recording for D1–D7), *N* = 24, ^NS^Not significant.

^∗^Significant (*p* ≤ 0.001).

**TABLE 8:** Post hoc test showing pair‐wise comparison of mean pretest & post‐test GCS scores from day 1 to day 7 in the experimental group.

Group	Time points	*M*_*D*_	SE_MD_	*p* value
Experimental	Pretest‐day 1 (M)	0.000	0.000	—
	Pretest‐day 1 (E)	0.000	0.000	—
	Pretest‐day 2 (M)	−0.25	0.396	0.53
	Pretest‐day 2 (E)	−0.83	0.39	0.05^∗^
	Pretest‐day 3 (M)	−3.29	0.44	0.001^∗^
	Pretest‐day 3 (E)	−5.17	1.78	0.01^∗^
	Pretest‐day 4 (M)	−3.88	0.44	0.001^∗^
	Pretest‐day 4 (E)	−4.04	0.45	0.001^∗^
	Pretest‐day 5 (M)	−4.33	0.47	0.001^∗^
	Pretest‐day 5 (E)	−4.33	0.47	0.001^∗^
	Pretest‐day 6 (M)	−4.92	0.56	0.001^∗^
	Pretest‐day 6 (E)	−5.00	0.55	0.001^∗^
	Pretest‐day 7 (M)	−4.50	0.68	0.001^∗^
	Pretest–Post‐test	−4.50	0.68	0.001^∗^

*Note:* ^NS^Not significant (*p* > 0.05).

^∗^Significant (*p* ≤ 0.05).

**TABLE 9:** RM‐ANOVA showing comparison of level of consciousness (GCS) before and after administration of multisensory stimulations from day 1 to day 7 in the comparison group.

Group	Time periods	Mean ± SD	*F* value	*p* value
Comparison	Pretest	6.92 ± 3.59	142.58	0.001^∗^
	Day‐1 (M)	6.92 ± 3.59		
	Day‐1 (E)	7.04 ± 3.53		
	Day‐2 (M)	6.92 ± 3.78		
	Day‐2 (E)	6.46 ± 3.43		
	Day‐3 (M)	10.23 ± 9.21		
	Day‐3 (E)	8.92 ± 4.25		
	Day‐4 (M)	9.42 ± 4.37		
	Day‐4 (E)	9.54 ± 4.35		
	Day‐5 (M)	10.12 ± 4.71		
	Day‐5 (E)	10.23 ± 4.62		
	Day‐6 (M)	8.85 ± 5.41		
	Day‐6 (E)	8.85 ± 5.41		
	Day‐7 (M)	8.92 ± 5.18		
	Post‐test	8.92 ± 5.18		

*Note:* From day 1 to day 7, the M&E value represents the average of GCS at an interval of every 15 min. Sample for RMANOVA was 26 as only 26 patients had GCS recording for d‐1 to d‐7, *N* = 26, ^NS^Not significant (*p* > 0.05).

^∗^Significant (*p* ≤ 0.05).

**TABLE 10:** Post hoc test showing pair‐wise comparison of mean pretest & post‐test GCS score from day 1 to day 7 in the comparison group.

Group	Time points	*M*_*D*_	SE_MD_	*p* value
Comparison	Pretest‐day 1 (M)	0.000	0.000	—
	Pretest‐day 1 (E)	−1.12	0.09	1.00^NS^
	Pretest‐day 2 (M)	0.000	0.56	0.47^NS^
	Pretest‐day 2 (E)	0.46	0.63	0.07^NS^
	Pretest‐day 3 (M)	−3.31	1.76	0.001^∗^
	Pretest‐day 3 (E)	−2.00	0.45	0.001^∗^
	Pretest‐day 4 (M)	−2.50	0.59	0.001^∗^
	Pretest‐day 4 (E)	−2.62	0.60	0.001^∗^
	Pretest‐day 5 (M)	−3.19	0.78	0.001^∗^
	Pretest‐day 5 (E)	−3.31	0.77	0.001^∗^
	Pretest‐day 6 (M)	−1.92	0.88	0.04^∗^
	Pretest‐day 6 (E)	−1.92	0.88	0.04^∗^
	Pretest‐day 7 (M)	−2.00	0.91	0.04^∗^
	Pretest–Post‐test	−2.00	0.92	0.04^∗^

*Note:* ^NS^Not significant (*p* > 0.05).

^∗^Significant (*p* ≤ 0.001).

The comparison group experienced a significantly higher incidence of tachycardia (33.03%) than the experimental group (12.69%) (*χ* ^2^ = 23.89, *p* = 0.001). Other adverse events, including bradycardia, hypotension, hypoglycemia, and hyperglycemia, showed no statistically significant differences between groups (*p* > 0.05) (Table 11). Tables 11 and 12 summarize the incidence of physiological adverse events.

**TABLE 11:** Comparison of incidence of serious physiological adverse events in the morning from day 1 to day 7 in the experimental and comparison groups.

PAEs	Group				*χ* ^2^	df	*p* value
	Comparison (*T* = 218)	*f* (%)	Experimental (*T* = 197)	*f* (%)			
*1. Bradycardia*							
Yes	15	6.88	0	0			
No	203	93.12	197	100			

*2. Tachycardia*							
Yes	72	33.03	25	12.69	23.89		0.001^∗^
No	146	66.97	172	87.31			

*3. Bradypnea*							
Yes	1	0.46	0	0			
No	217	99.54	197	100			

*4. Tachypnea*							
Yes	4	1.83	2	1.02	0.49		0.49^NS^
No	214	98.17	195	98.98			

*5. Hypotension*							
Yes	6	2.75	2	1.02	1.65		1.99^NS^
No	212	97.25	195	98.98			

*6. Hypertension*							
Yes	67	30.73	46	23.35	2.84		0.09^NS^
No	151	69.27	151	76.65			

*7. Hypoglycemia*							
Yes	1	0.46	2	1.02	0.45		0.50^NS^
No	217	99.54	195	98.98			

*8. Hyperglycemia*							
Yes	29	13.30	32	16.24	0.71		0.39^NS^
No	189	86.69	165	83.75			

*9. Hypoxia*							
Yes	11	5.05	10	5.08	0.0002		0.99^NS^
No	207	94.95	187	94.92			

*Note:* ^NS^Not significant (*p* > 0.05).

^∗^Significant (*p* ≤ 0.05).

**TABLE 12:** Comparison of incidence of serious physiological adverse events in the evening from day 1 to day 7 in the experimental and comparison groups.

PAEs	Group				*χ* ^2^	df	*p* value
	Comparison		Experimental				
	(*T* = 218)	*f* (%)	(*T* = 197)	*f* (%)			
*Tachycardia*							
Yes	74	33.9	22	11.17	30.2		0.01^∗^
No	144	66.1	175	88.8			

*Bradycardia*							
Yes	14	6.4	3	1.5	6.3		0.01^∗^
No	204	93.6	194	98.5			

*Tachypnea*							
Yes	5	2.3	1	0.5	2.31		0.13^NS^
No	213	97.7	196	99.5			

*Hypotension*							
Yes	7	3.2	4	2.0	0.55		0.45^NS^
No	211	96.8	193	97.9			

*Hypertension*							
Yes	23	10.5	24	12.2	0.28		0.60^NS^
No	195	89.4	173	87.8			

*Hypothermia*							
Yes	1	0.5	1	0.5	0.005		0.94^NS^
No	217	99.5	196	99.5			

*Hyperthermia*							
Yes	1	0.5	0	0			
No	217	99.5	197	100			

*Hypoglycemia*							
Yes	2	0.9	0	0			
No	216	99.1	197	100			

*hyperglycemia*							
Yes	35	16.1	25	12.7	0.94		0.33^NS^
No	183	83.9	172	87.3			

*Hypoxemia*							
Yes	8	3.7	14	7.1	2.43		0.11^NS^
No	210	96.3	183	92.9			

*Note:* ^NS^Not significant (*p* > 0.05).

^∗^Significant (*p* ≤ 0.05).

## 4. Discussion

Multisensory stimulation might have a vital role in holistic patient care by reducing complication while ICU stay stemming from prolonged immobility and sensory deprivation. The coalescence of auditory and tactile interventions used in the study mirrors the therapeutic practicality of integrating familiar, comforting stimuli often delivered by bloodline members to assist patients and stimulate neurocognitive engagement. This lines up with the postulates of neuroplasticity, particularly beneficial in conditions like stroke, traumatic brain injury, and hypoxic encephalopathy. Further, it is bound with emotionally remarkable involvement of family may act synergistically to improve the outcomes of patient, offering combined benefits of physiological and psychosocial recovery trajectory.

The present study evaluated the efficacy of multisensory stimulation in the level of consciousness and physiological parameters in the altered consciousness patients admitted to ICU. The intervention was administered by the patient’s caregiver and the researcher. The hypothesis was that there would be a significant difference in the levels of consciousness of the patients with altered states of consciousness, and in the frequency of physiological adverse events after administration of multisensory stimulation between the experimental and the comparison groups. The present study also observed that most of the participants in both groups were between the ages of 60 and 87 years. This finding suggests that older patients are likely to be more vulnerable to longer unconsciousness, thus making them more vulnerable to sensory deprivation. These findings are in agreement with previous studies, which have also reported that unconscious patients are more likely to fall within the age range of 40 years and above, with age groups extending up to 71–91 years [12].

In this study, a large proportion of participants of the comparison (59.5%) and experimental (44.1%) groups had already been diagnosed with neurological illnesses of CVA, SDH, and meningitis. These illnesses cause alterations in levels of consciousness and hence necessitate interventions like multisensory stimulation. Besides neurological illnesses, intensive care unit patients in the experimental and comparison groups were also diagnosed with renal (18.9% vs. 23.5%) and cardiothoracic (18.9% vs. 17.6%) disorders. These patients can also experience alterations in consciousness from diseases like hypoxia, metabolic derangement, hemodynamic instability, or extended ICU stay, which resulted in sensory deprivation [13].

Although multisensory stimulation in individuals experiencing brain injury–related impairments has been explored extensively, its application to other critically ill patient populations is as yet not exhaustively investigated. The dominance of TBI literature identifies a knowledge gap in the impact of multisensory stimulation on recovery among patients affected by different underlying conditions. In the present study, such variables as neurological, cardiothoracic, and renal illness may have affected heterogeneity of patient response to the intervention and therefore would make it difficult to identify its specific effect. The limited research studies quantifying multisensory stimulation in more than a single diagnostic group present a formidable challenge in establishing its efficacy over a range of critically ill populations.

Male participants comprised the majority in both groups, with 55.8% in the experimental and 62.2% in the comparison group, as observed in the current investigation. These results are consistent with results in previous studies [9], where the same male dominance was observed in patients who received tactile stimulation to increase consciousness and vital signs in patients with traumatic brain injury, as the comparison cohort had a ratio of 90%–10% and the experimental cohort had a ratio of 78.3%–21.7%. On the other hand, another research [14] on coma arousal technique effects on unconscious patientsȁ clinical outcomes showed that over half of the study samples were female in the experimental group (57% vs. 43%) and comparison group (63% vs. 37%). Repetitive stimuli were also to be instrumental in stimulating consciousness of patients. A statistically significant difference was found between the level of consciousness in the experimental group (12.89 ± 3.53) and comparison group (9.10 ± 4.51) on the fourth morning (*t* = −3.51, *p* = 0.001) in the present study. This concurs with findings in a previous study where there was a notable difference between experimental group average scores of consciousness (5.73 ± 0.90) and the control group (5.13 ± 1.00) on the third day following auditory stimulation (*t* = 2.42, *p* = 0.019). The findings of the present study indicated a statistically significant alteration in both the groups (*p* = 0.001). The result agrees with another study [15] which shows that the significant mean GCS score was found in both the groups (*p* = 0.0001). Mandeep et al. [13] conducted a study, which revealed the statistically significant improvement on day 7 of GCS (*p* = 0.001). Moattari et al.’s [15] study findings indicated increased levels of patients’ consciousness during a 7‐day intervention period. The research also documented higher improvement levels when intervention was through family members, suggesting higher intervention in cognitive and sensory recovery by family involvement. The intervention employed a wide, multimodal intervention and sensory stimulation such as visual, tactile, and olfactory cues [16]. Some research studies suggest that a minimum of 2 weeks is usually the shortest period of time that is normally required to facilitate notable increases in levels of consciousness. Similar to Mohammadi et al. [17] also revealed that the intervention group exhibited a statistically significant improvement in mean GCS scores on the 14th day of study (*p* = 0.001). Oh and Seo’s [16] research findings revealed better results after 2 weeks of intervention, highlighting the significance of having a minimum intervention period of 2 weeks to look for good outcomes. Moghaddam et al. [18] study’s findings further demonstrated that sensory stimulation effects could only become evident after at least 2 weeks, once more supporting the therapeutic value of such interventions in promoting recovery. This helps to underpin the highest demand for early, specific, nonpharmacological interventions, e.g., multisensory stimulation, to reverse sensory deprivation and enable neurological recovery [18]. Multisensory stimulation could be a key to supporting sensory pathways, increasing cortical activation, and preserving neuroplasticity even in patients with decreased consciousness. This intervention could provide continuous sensory stimulation if given reliably by caregivers and healthcare professionals. It has the potential to provide ongoing sensory input that helps stimulate cognition, accelerating recovery and reducing the risk of persistent cognitive and physical impairment [15]. Furthermore, these findings would warrant the development of age‐adjusted, individualized intervention strategies for the treatment of the unique physiological and cognitive needs of elderly ICU patients The addition of multisensory stimulation to routine critical care protocol could potentially have the added benefit of not only increasing the level of consciousness and physiological stability but enhancing also patient comfort and emotional status, so often overlooked in traditional ICU practice.

Through widening the evidence base and making patient‐centered, nonpharmacological interventions a high priority, health systems are more able to meet the multidimensional needs of unconscious, older patients and ultimately achieve better outcomes and quality of life in this vulnerable group. A study result by Mohammadi et al. [19] revealed statistically significant differences in body temperature and blood pressure. YekeFallah in another study, found significant differences in vital signs among the patients in the experimental group following the administration of auditory stimulation with music and tape‐recording voice messages from their relatives. Yekefallah et al. studied the impact of tactile stimulation on consciousness and vital signs in head‐injured patient, and also documented significant reduction in heart rate, SBP, DBP, and respiratory rate (*p* ≤ 0.05), whereas there were no changes in interventional group’s body temperature. Nonetheless, research laid stress on enhanced level of consciousness and stable vital signs [12]. The outcome of the study reveals that multisensory stimulation did not result in adverse physiological impacts when the patient parameters were measured over the experimental and comparison groups. This implies that the intervention is a safe method of stimulating neurocognitive recovery without undermining physiological stability. However, the comparison group exhibited a significantly higher incidence of physiological complications, including tachycardia, bradycardia, hypertension, hyperglycemia, and hypoxemia. This suggests that multisensory stimulation may contribute to stabilizing physiological parameters, potentially reducing the occurrence of such adverse events. The study is consistent with prior research. For instance, Mohammadi et al. [17] examined the impact of the coma arousal technique on unconscious patients and report no harmful physiological effects. Instead, key physiological measures such as RR, HR, BP (systolic and diastolic), oxygen saturation, and blood glucose levels demonstrated notable improvement and maintained stability. This stability may result from the technique’s role in promoting equilibrium between the sympathetic and the sympathetic nervous systems. Similarly, Hoseini et al. [20] reported that the implementation of auditory stimulation did not result in any adverse effects among coma patients. In the present study, adverse events varied across different times of the day, with notable differences between morning and evening assessments. The findings of morning time revealed that physiological adverse events were notably increased in the comparison group than in the experimental group. Specifically, the incidence of bradycardia (6.88% vs. 0%), tachycardia (33.03% vs. 12.69%), and hypertension (30.73% vs. 23.35%) was more commonly reported in the comparison group. Conversely, hyperglycemia was more frequently observed in the experimental group (16.24%) compared to the comparison group (13.30%). Results of evening time revealed that physiological adverse events were more prevalent in the comparison group than in the experimental group. Specifically, the incidence of bradycardia (14% vs. 3%), tachycardia (74% vs. 22%), tachypnea (5% vs. 1%), and hyperglycemia (35% vs. 25%) occurred more frequently in the comparison group. Conversely, hypoxia was more frequently observed in the experimental group (14%) compared to the comparison group (8%). Results of the investigations are consistent with the study by Ahmed et al., which suggested that the intervention group demonstrated better stability of physiological parameters as compared to the experimental group with regard to respiratory rate, heart rate, SpO_2_, and blood glucose level [6]. On the other hand, Mousa Sajjadi study indicated that there were no statistically significant differences in the physiological parameters of blood pressure, body temperature, and SpO_2_, both prior to and following the administration of the intervention [21]. The variability in study outcomes may be recognized to differences in study duration, the intensity and type of stimulation provided, level of consciousness among participants, the presence of multifaceted diagnoses, and the involvement of family caregivers. These factors could influence the effectiveness of the intervention, potentially explaining why some studies did not observe significant changes in physiological parameters. This highlights the need for further research to explore the specific conditions under which multisensory stimulation may have a measurable impact on patient outcomes.

### 4.1. Limitations

The limitation of the study is that it lacks randomization, which may have introduced selection bias and limit the ability to control for confounding variables. Given the limited sample size and the study’s confinement to a single setting, the findings may not be generalizable to the wider population. Moreover, patient length of stay was not examined as part of this investigation.

## 5. Conclusion

The analysis conducted herein suggests that the use of multisensory stimulation led to improvements in the patients′ levels of consciousness and a reduction in the incidence of adverse physiological events. As with ending, the study offers compelling evidence that multisensory stimulation is a safe, noninvasive, and effective nursing intervention for improving consciousness and stabilizing the physiological parameters in ICU patients with altered consciousness. Given its grounded nature, cost‐effectiveness, and potential for integration into routine care, MSS should be reached out as a standard adjunct therapy under rehabilitative management in critical care settings.

Future research based on this should pursue to expand the sample size, adopt randomized controlled designs, and scrutinize long‐term outcomes such as cognitive function, length of ICU stays, and wholesome approaches of survival to further validate and generalize these findings.

## Funding

No funding was received for this research.

## Ethics Statement

The ethical approval was obtained from Institutional Ethical Committee of Maharishi Markandeshwar (Deemed to be) University. Mullana, Ambala, Haryana.

Formal administrative approval was obtained from Medical Superintendent of the hospital.

## Consent

An informed consent was attained from the patients’ relatives on behalf of the patients.

## Conflicts of Interest

The authors declare no conflicts of interest.

## Data Availability

The data that support the findings of this study are available upon request from the corresponding author. The data are not publicly available due to privacy or ethical restrictions.
